# Development of an online service for coping with spousal loss by means of human-centered and stakeholder-inclusive design: the case of LEAVES

**DOI:** 10.1080/07481187.2023.2203680

**Published:** 2023-04-27

**Authors:** Lex van Velsen, Lotte Schokking, Eva Siderakis, Gloria-Mona Knospe, Lena Brandl, Bettina Mooser, Sarah Madörin, Sofia Jacinto, Afonso Gouveia, Jeannette Brodbeck

**Affiliations:** a eHealth group, Roessingh Research and Development, Enschede, Netherlands; b National Foundation for the Elderly, Amersfoort, Netherlands; c NOTHING AG, Köniz, Switzerland; d Department of Human Media Interaction, University of Twente, Enschede, Netherlands; e Department for Clinical Psychology and Psychotherapy, University of Bern, Bern, Switzerland; f School of Social Work, University of Applied Sciences and Arts Northwestern Switzerland, Olten, Switzerland; g Iscte-Instituto Universitário de Lisboa, CIS-Iscte, Lisboa, Portugal; h Psychiatric Department at The Health Unit of Baixo Alentejo, Beja, Portugal

## Abstract

To support older mourners after the loss of their partner, LEAVES, an online self-help service that delivers the LIVIA spousal bereavement intervention, was developed. It integrates an embodied conversational agent and an initial risk assessment. Based on an iterative, human-centered, and stakeholder inclusive approach, interviews with older mourners and focus groups with stakeholders were conducted to understand their perspective on grief and on using LEAVES. Subsequently, the resulting technology and service model were evaluated by means of interviews, focus groups, and an online survey. While digital literacy remains a challenge, LEAVES shows promise of being supportive to the targeted end-users.

## Introduction

The loss of a partner is a life event with high impact that occurs to many older adults and grief is a normal reaction to this loss (Holland et al., 2009). While most mourners can process this grief and develop a new life without the partner, a subset of people struggles and develops a Prolonged Grief Disorder (PGD). To prevent PGD, several online self-help interventions and eHealth services have been developed and evaluated in randomized controlled trials. The implementation of eHealth services may benefit from a service model, which consists on the description of all the steps that an end-user takes when using the service, while also displaying the activities that other stakeholders perform concerning the service and the end-user’s journey (Bitner et al., 2008). As such, a service model provides important input for eHealth design and implementation plans.

The present paper focuses on the development and implementation of LEAVES, an eHealth service to support the grief process and prevent PGD of older adults who lost their partners. PGD is the failure to return to pre-loss levels of performance or wellbeing (Prigerson et al., 1995). Symptoms that are associated with PGD include separation distress (like emotional pain), reactive distress (like self-blame), and social/identity disruption (like loneliness). A meta-analysis of 14 studies revealed a pooled prevalence of PGD of 9.8%, with a higher risk in older age (Lundorff et al., 2017).

Several online interventions for treating or preventing PGD have been evaluated. A recent systematic review of the efficacy of online interventions for grief after bereavement included nine trials and identified effects of these services on symptoms of grief, depression, and post-traumatic stress (Zuelke et al., 2021). Furthermore, the authors concluded that the quality of the interventions (that were, with one exception, all based on cognitive behavioral therapy) was high. Of special interest for this study is the LIVIA intervention, an online self-help program that supports older adults with spousal bereavement. It is based on the task model of mourning (Worden, 2009) and the dual-process model of coping with bereavement (Stroebe & Schut, 1999). It consists of ten text-based modules that are focused on information about interpersonal loss and an assessment of the current personal situation, exposure, and loss-oriented interventions for accepting memories and pain and addressing unfinished business, as well as resources and restoration-oriented interventions for fostering positive emotions, self-care, positive social relationships. and creating a new life without the partner. In a randomized controlled trial (Brodbeck et al., 2019, 2022), LIVIA was found to improve grief, depressive symptoms, psychopathological distress, embitterment, loneliness, and life satisfaction.

In the LEAVES project (optimizing the mentaL health and resiliencE of older Adults that have lost thEir spouSe via blended, online therapy) (van Velsen et al., 2020), LIVIA forms the basis of a new online service for coping with the loss of a partner at a later age. The texts that form the basis of LIVIA were translated into dialogues that are presented to the mourner via an Embodied Conversational Agent (ECA). An ECA is a virtual entity that interacts with the end-user through technology, for example via predefined answers which the end-user can choose from. For the context of mental health care, ECAs have been found to improve therapeutic alliance (Suganuma et al., 2018) and thus, to increase compliance with the service and the intervention it provides. A review study found that ECA interventions can treat mild to moderate psychological distress in adults (Gaffney et al., 2019). LEAVES’ ECA is intended to guide the user through the service and to introduce and wrap up the work the user does within the intervention modules. This ECA also guides users through the suggested practical activities and the functionalities dedicated to help the users checking their own mood and to remind them of their best strategies to cope with grief or to seek further support.

Apart from the ECA, an initial risk assessment has been added to the content of LIVIA. When first using LEAVES, mourners must take a risk assessment that determines whether they are recommended to use the service or seek further in-person professional help. Moreover, while using the program, a bi-weekly monitoring gives further recommendations for seeking additional help if necessary. The monitoring is based on mental health user profiles informed by a three-round Delphi study involving clinical experts and stakeholders, described in Brandl et al. (2022).

In this article, we discuss the development of the LEAVES service and the corresponding service model. Both human-centered and stakeholder-inclusive design principles were key to the development of LEAVES. Therefore, prospective end-users were actively involved in deciding upon the design of the service, while stakeholders (e.g., general practitioners, funeral homes) were consulted regarding the role they foresee to play in the service provision. Stakeholders consist of any group of people or organizations that have a professional task, or role, related, or affected, by a service that is not directly used by the said stakeholder (Van Velsen et al., 2013). Including their views and wishes on the future service is very important to ensure that the service will run as expected in the designated context of use (Broekhuis et al., 2021).

The application of these design approaches was found to increase the usefulness and chances of success for eHealth services (Oberschmidt et al., 2020; Van Velsen et al., 2013). However, reports of these development approaches for services like LEAVES are scarce, while sharing them is paramount for enabling learning and improving the quality of these services in general (Kramer et al., 2020). By presenting the LEAVES development process and sharing its outcomes, this article aims to inform other developers and implementation specialists on the lessons we learned, while showcasing the possibilities of an eHealth tool directed at older mourners to the healthcare community.

## Methods

The development of LEAVES was an iterative process whereby input was collected from potential end-users (mourning older adults), care professionals, and other stakeholders. The process has four main phases: understanding older adult mourners, their context, and their grief journey; discussing stakeholders’ roles and perspectives in the LEAVES service; discussing the first impression and potential of LEAVES with end-users; and validating the LEAVES design, the service model, and the implementation strategy. Since the LEAVES service was designed to be implemented in three countries (The Netherlands, Switzerland, and Portugal), all design activities were held within these countries. For all the studies described, the materials were developed in English and translated to Dutch, German, and European Portuguese, to be used in each implementation country.

The study was carried out in accordance with the Declaration of Helsinki. Written informed consent was obtained from each participant. The nature of this general focus group and interview study with the focus on service model development among voluntary participants did not require formal ethical approval, according to Dutch and Swiss laws.

### Understanding older mourners

One-on-one interviews were held with adult mourners to uncover how the LEAVES service can help them. Primarily, the goal was to learn about how they tried to cope with their grief. Secondly, this information was used to create an end-user persona: a fictitious end-user, representative of the group of people that were most likely to use LEAVES. Personas are most often presented in the form of a profile with a photo, describing their characteristics and attitude toward a service. They can be used as a reference during the design phase to ensure a focus on the potential end-user throughout the design process (LeRouge et al., 2013).

Semi-structured interviews were conducted with older mourners, in which we inventoried demographics and addressed the following topics: a description of their experienced grief journey; the struggles they encountered during their grief journey; the incentives they felt during their grief journey toward taking action; and the sources of help they sought for supporting their grief journey.

In this, and in all the studies described, participants were recruited via the professional networks of the researchers involved and provided informed consent before participating in the study. The interviews were conducted in person or via phone (depending on the applicable COVID-19 measures at a given time and place) and were recorded and transcribed. Data collected in the interviews were categorized according to the main research questions. Following this categorization, frequencies per country were obtained. Final results consisted of the total frequencies combining the three implementation countries. These results were further analyzed in two workshops to refine the initially proposed service model and the personas. In these workshops, the experiences of the interviewers and the non- and paraverbal information during the interviews served as important input for the interpretation of the results.

### Stakeholders’ roles and perspectives in the LEAVES service

We conducted several focus group sessions with representatives from the most important stakeholders for LEAVES in the Netherlands, Switzerland, and Portugal. In these focus groups, the following topics were addressed: (1) Current grief-related service provision to mourners such as psychological grief counseling, pastoral care and community services, as well as the essential and missing elements of that service, considering both the loss-oriented and the restoration-oriented tasks proposed by the dual process model (Stroebe & Schut, 1999); (2) The added value that LEAVES could provide to mourners (relative to the current services available); (3) The role that stakeholders would like to play in the context of LEAVES; and (4) The potential barriers that stakeholders see for end-users of LEAVES. All focus groups were analyzed via an online workshop in which the researchers, collaboratively, grouped participants’ answers based on the topics’ frequency.

### The potential of LEAVES for end-users

To gauge potential end-users’ first reactions to grief support via an online service and to fill in the gaps in the service model designed, older mourners were consulted. To understand how end-users perceive the service interface and the virtual agent, a short demo version of LEAVES was presented to potential users while assessing their attitudes about an online service for coping with grief. The following topics were discussed: first reactions toward the LEAVES concept; first reactions toward specific functionalities of LEAVES (e.g., the ECA, the Notebook, and Activities section); advantages and disadvantages of online mental health support; and the roles that online and offline support can play during older adults’ grief journey.

### Validating the LEAVES design and the service model

The final stage of the design process entailed validating the service model with potential end-users and stakeholders. To assess their perceptions about the LEAVES service model, a quantitative online survey was developed. Each part of the survey was introduced by a short explanatory text and a relevant screenshot of the service. Separate surveys were created for end-users and stakeholders. In the survey, besides collecting the demographics, the following topics were addressed by means of closed questions: digital skills; importance of the LEAVES risk assessment; appropriate persons for supporting mourners at risk; the role of mental health monitoring; usefulness of LEAVES; added value of LEAVES for processing spousal loss (end-users) or professional tasks (stakeholders); and intention to use. All data were analyzed descriptively.

## Results

### Understanding adult mourners

In the three countries, 23 adult mourners participated in the interviews. Their demographics can be found in Supplementary Table 1. When describing their grief journey, participants mainly applied one of two coping strategies. Some mourners applied an active strategy. They had a plan in mind and actively sought ways to cope with grief and to rebuild their lives. They searched for new activities, tried to develop a new rhythm in their lives, read about the mourning process, or talked to other people about their loss. Other mourners applied a passive strategy, letting their grief run unchecked, as epitomized by the image of “just sitting in a chair and staring out of the window, not knowing what to do.”

**Table 1.** Most important elements of grieving support services.

Dual process phase	Essential and currently available	Essential, but currently not available
Loss-oriented	Exchanging grief experiences with peers Providing purpose in life Informing family and friends about the process of grief Caregivers being able to understand the mourners and their strengths (before the spousal loss) Keeping objects to remember the deceased spouse	Addressing grief in the social environment Prevention of social isolation Allowing mourners to keep talking about their partner and their grief.
Restoration-oriented	Preparing for the situation after the loss (practically) Exchanging experiences with people who are in different phases Assistance with topics of shame, especially finances Making lists of new things they want to learn Connecting with family and friends Practical tools and action lists A good relationship with the general practitioner Connecting with different caregivers A monitoring function	Improving mourners’ knowledge about coping with grief Help in building up social networks Establishing contact with professional contact points Actively approaching the bereaved Acquiring emotional, social, and daily living skills Communicating about upcoming activities Supporting volunteer work Informing about support options Offering different types of activities to cater for personal differences

The main reasons why participants sought additional help were mental challenges, like excessive worrying, lack of motivation, or anxiety (mentioned 10 times). Next in order, a lack of social relationships since losing the partner was mentioned (9 times), followed by physical challenges, like headaches and sleeping problems (mentioned 8 times). Most of the participants who did seek help did so either after 6 months (5 interviewees), after a year or within the first two months after the loss (both mentioned by 3 interviewees). The decision to use additional help was mostly initiated either by the mourners themselves (12 interviewees), by friends or relatives (9 times), or by medical professionals (8 times).

The search for help was mostly conducted locally and solutions were most often also found nearby. Being able to talk to a person face-to-face was deemed very important. Initially, their search was focused on the actors they were already in contact with, such as the general practitioner or the hospital. Few sought help via the internet. Interviewees found that it was important to have external help that was easy to access, that allowed them to help others as well, and provided them with the opportunity to improve their relationship with family members.

With the input of the interviews, a persona was defined, *Monica*, who lost her husband and, as a result, is feeling lonely. She is open to help, as long as she feels that she can trust this kind of help. She does not want to bother her social circle with her ‘grieving problems’ and is therefore seeking a personal, private safe space.

### Stakeholders’ roles and perspectives in the LEAVES service

In the three countries, 23 stakeholders participated in the focus group sessions. Their characteristics are described in Supplementary Table 2. The participants reflected on the way in which grief is dealt with in the different countries. In the Netherlands, participants were primarily active in primary care or in social roles, while in Switzerland, the hospital and the church played a larger role. A more medicalized outlook is also present in Portugal, with the presence of a geronto-psychiatric nurse, a psychiatrist, and a general practitioner.

**Table 2.** Results of the stakeholder survey for validating the service model.

	Netherlands		Portugal		Switzerland	
	*N*	*%*	*n*	*%*	*n*	*%*
*Necessary digital skills for onboarding procedures:* End-user can use the onboarding						
Most or all of the older adults can	17	41.5	5	22.7	4	14.8
Most cannot	15	36.6	12	54.5	16	59.3
None can	2	4.9	1	4.5	1	3.7
I don’t know	7	17.1	4	18.2	6	22.2
*Support in times of need*						
Friend, family member	27	61.4	17	65.4	20	69.0
Peer, self-help group	17	38.6	13	50.0	8	27.6
Psychologist	7	15.9	11	42.3	9	31.0
Church	16	36.4	4	15.4	12	41.4
General practitioner	7	15.9	7	26.9	9	31.0
Social worker	14	31.8	2	7.7	6	20.7"
	*M*	*SD*	*M*	*SD*	*M*	*SD*
*Importance of risk assessment* ^a^	4.25	.81	4.32	.72	3.72	1.14
*Trust in the monitoring function* ^b^	3.06	.90	3.47	.80	3.41	1.22
*Acceptance of LEAVES* ^c^						
LEAVES will be easy to use	3.00	.76	3.35	.61	3.45	.60
LEAVES will be useful	3.59	.88	3.71	.77	3.41	.85
LEAVES could be effective for some of my clients	3.44	.98	3.59	.71	3.32	.78
LEAVES would be useful as a stand-alone tool	2.58	1.03	3.24	.97	2.73	.88
LEAVES would be useful in blended counseling	3.75	.88	3.88	.78	3.57	.87
I intend to use LEAVES in my work	3.03	.97	3.29	1.16	3.41	.80

Note: ^a^Response options from 1 = very unimportant to 5 = very important. ^b^Response options from 1 = not to 5 = a lot. ^c^Response options from 1 = totally disagree to 5 = totally agree.

Data analysis resulted in an inventory with the participants’ perception on the essential and missing elements of current services for supporting coping with grief. The most important elements are provided in Table 1. There were also some country-specific elements that needed to be considered. In the Netherlands, stakeholders emphasized that the country, as individualistic society, provide services that focus on enabling mourners to get on with their lives, neglecting the mourners’ need to talk about the deceased. A tailored service was considered essential. In Switzerland, the lack of time for formal caregivers such as general practitioners poses a major problem for properly dealing with grief and seeking for a comprehensive solution. Then, social isolation associated with aging is considered to be a challenge to promote further new connections with other people. In Portugal, the geographic and social isolation that many older adults face play an important role when dealing with grief. At the same time, mourning is considered to be a social activity where the mourner’s family and religion play a large role. In Portugal as well as in Switzerland and the Netherlands, stakeholders note that families tend to move closer together during times of mourning and provide immediate and practical support. However, formal and informal carers’ tendency to avoid talking about the deceased or the grieving process after the acute phase raises the need for further emotional support. While literacy on the topic of grief is low in Portugal, the service should allow for the possibility to learn about it. Associated to the low digital proficiency, ownership of technology, such as smartphones, tablets or laptops is low.

After being introduced to the concept of the LEAVES service, the stakeholders mentioned that the service could support them in providing psychoeducation on the topic of grief (possibly in combination with exercises). Moreover, the flexibility of the service was praised, as it allows mourners to have control of their grief process, by working on their grief at their own pace, and increases health access, since mourners are not restricted to the office hours of care professionals. Furthermore, the stakeholders mentioned that LEAVES could act like a hub and connect different activities, services, and resources. The Portuguese stakeholders commented on the fact that LEAVES can monitor the mental health of mourners, both for informing the mourner and the stakeholders themselves. Finally, in the Netherlands, LEAVES was considered to be a service that can be beneficial for the family of the mourner, as it can provide them with information on how to deal with the situation.

Regarding referring clients to LEAVES, the stakeholders agreed that this can only happen if the mourners are motivated and stable enough to make progress in their processing of grief on their own. Moreover, stakeholders also mentioned that the lack of human warmth of online services may not seem suitable or attractive to be used as a self-service technology. These requirements were voiced primarily in Switzerland and Portugal. In the Netherlands, the opposite was stated. LEAVES was seen as a safe space, as mourners can interact with the service in privacy and from the comfort of home. Stakeholders in all countries highlighted the need for empirical evidence for the efficacy of LEAVES and the importance of the combination with in-person support. They emphasized that LEAVES should be integrated into an ongoing treatment or could serve as a starting point for later in-person counseling.

The stakeholders identified several barriers for using LEAVES, such as low digital literacy, an aversion to or fear of digital services and a lack of assistance for using these types of services from the social network. Finally, the Dutch stakeholders thought it would be difficult to monitor the mental health of the mourner via an online service only. To overcome the disadvantages of an online self-help service, stakeholders suggested that people from the social network of the mourners, like relatives, could be involved and have a role in the LEAVES service. To counteract the issues that derive from low digital literacy, an easy and intuitive interface, in combination with a tutorial or introduction video, could be used, as well as allowing mourners to download the intervention in a book form or to blend the service with face-to-face counseling.

In sum, the interviews with potential end-users and focus groups with stakeholders allowed the development of an initial service model for LEAVES. The service model specifies that older adult mourners with mental, social, physical, or practical challenges (e.g., financial difficulties) visit a general practitioner, church or peer group that points them to LEAVES. Alternatively, older mourners can be steered toward the service by a municipality, undertaker, family, or friends, or find it as the result of an online search. When first using LEAVES, mourners will have to take a risk assessment that determines whether the end-user can process the loss with the help of LEAVES or needs in-person professional help. If mourners are approved for use, they will go through an online tutorial that explains the use of the service. Then, mourners can enter the contact details of persons that can help them in times of need (on the assumption that people in psychological distress find it difficult to think rationally and come up with potential helpers) and who will be reminded when additional help is necessary. The mourners can then work through the different LEAVES modules. Every two weeks, the mourners are asked to complete an online questionnaire that assesses their mental state. If this assessment indicates high emotional distress, mourners are invited to seek professional help. Otherwise, the mourners are expected to complete the ten LEAVES modules, after which the service is completed.

The development of the service model led to four questions subsequent questions: In each country, which party could offer the service? When does LEAVES need to be changed from an online into a blended service? Where do mourners go if they are not approved to use LEAVES? With regard to the contact list, what type of notifications are given to the mourner, how does the contact list interact with the assessment of the mourner’s mental state, and who decides which person is contacted and when?

### End-users’ first reactions to the LEAVES concept

In the three countries, 16 older adults who have not taken part in previous interviews or focus groups participated in either an interview or focus group. Their demographics are displayed in Supplementary Table 3. Results show that none of the participants had used online resources when dealing with their grief. Regarding receiving online help from LEAVES, the participants were, in general, curious and open to give it a try. However, they mentioned that online help could never replace in-person professional counseling. They saw the main advantages of LEAVES in the possibilities of obtaining a wider range of information about the grief process and how to adapt to a world without the deceased, a lower threshold to start, the anonymity, and its convenience (i.e., the independence of time and place). On the other hand, low digital literacy was mentioned multiple times as an inhibiting factor. Asked about the online Notebook, one of the main functionalities in LEAVES, some participants stated that they would use the option to write in an online grief diary (“to get things out of my head”), while others stated that they would not do so at all, or instead would prefer pen and paper.

The main sources of offline help for mourners were either family and friends or the general practitioner, followed by peer groups, counselors, or therapists. Participants considered these sources of support as very valuable while processing their loss. Additionally, keeping themselves busy by means of sports or hobbies (e.g., gardening, meditation) was considered very helpful.

With the help of these insights, we finalized the concept service model. Adding to what was defined in the previous phase, it was defined that the mourners can learn about the service via care professionals, their social network, or by searching for it themselves. Throughout the LEAVES journey, the end-users will be guided through the service by the embodied virtual agent. In an iterative process between the development of the service model and the design team, it was defined that the LEAVES’ ECA should be an abstract entity (as opposed to a human-like entity) which would be called Sun.

### Validating the service model

In the three countries, 99 persons completed the online survey for stakeholders; 44.4% lived in the Netherlands, 29.3% in Switzerland and 26.3% in Portugal. Mean age was 49 years (SD = 12.45); 77 were women (77.8%) and 22 (22.2) were men. In the Netherlands, the majority of participants worked in spiritual care (e.g., as spiritual counselor or church employee; n = 18, 40.9%) or in health care (e.g., as psychiatrist or nurse; n = 8, 18.2%). In Portugal, all 26 participants worked in health care. In Switzerland, about half (n = 16, 55.2%) worked in health care, about one third in spiritual care (n = 9, 31%).

In addition, 17 older mourners filled out an online survey for end-users, 11 (83.3%) from the Netherlands and 5 (16.7%) from Portugal. Mean age was 74 years (SD = 10.79); 11 were women (64.7%), 6 were men (35.3%).

After showing participants the onboarding procedure, participants were asked whether they thought that the prospective end-users had the necessary digital skills to complete this part of LEAVES. In the Netherlands, 91.7% of the older adult mourners thought they would be able to, while in Portugal 75.0% thought so. Asking the stakeholders the same question, only 41.5% of the Dutch stakeholders, 22.7% of the Portuguese and 14.8% of the Swiss stakeholders thought that most or all of the older adults would be able to complete this task. Detailed results are presented in Table 2.

In all countries, family members or friends were thought of as the most suitable person to help older adults when using LEAVES (77.3% in the Netherlands, 72.4% in Switzerland, and 65.4% in Portugal). Additionally, in Portugal 42.3% of the participants expected the caregiver to take on this role.

Next, we showed stakeholders the LEAVES risk assessment that determines whether the mourners are recommended to use the service or seek further in-person professional help. In the Netherlands and Portugal, stakeholders on average rated the risk assessment as important to very important, in Switzerland, as neutral to important. Among the Dutch and Portuguese stakeholders, about 45% thought this feature was very important, while Swiss stakeholders only 28.0% thought this was very important.

At this point, we showed the participants the section in LEAVES where they are asked to list some persons who they could contact in times of need. Most participants mentioned a friend or family member, followed by a peer (another mourner, member of a self-help group), someone from the church, a social worker, a psychologist, or the general practitioner. Friends and family members were mentioned most often in all countries. In the Netherlands, peers and the church were important as contact in times of needs; in Portugal, peers and psychologists were often mentioned and in Switzerland, the church was often mentioned (see Table 2).

The remaining questions focused on participants’ acceptance of the LEAVES service. Stakeholders were asked if they would trust the LEAVES to estimate and monitor the mourner’s mental health. In the Netherlands, 39.4% of the stakeholders thought they could trust this somewhat, 30.3% remained neutral, and 27.3% thought it would not really be able to do so. In Switzerland, 18.2% were sure to trust LEAVES with this task, 31.8% trusted this somewhat, and 36.4% remained neutral. In Portugal, finally, 5.9% trusted this functionality a lot, 47.1% somewhat, and 25.3% remained neutral. Means and standard deviations are presented in Table 2, along with the stakeholders’ reactions to several items assessing their acceptance of LEAVES.

The stakeholders had a neutral expectation toward its usability in the Netherlands, and a slightly positive expectation in Switzerland and Portugal. Regarding the usefulness and the potential effectiveness of the service for older mourners, the stakeholders were slightly positive in all countries. Noteworthy is that the stakeholders were neutral or slightly negative toward the use of LEAVES as a standalone tool, but (somewhat) positive about using LEAVES as part of blended counseling. The intention to use LEAVES in the stakeholders’ own work setting remained neutral (the Netherlands) or was slightly positive (Switzerland and Portugal).

## Discussion

In this article, we presented the design process of LEAVES, an online service that supports older adults in coping with the loss of a partner. The design process was human-centered and stakeholder inclusive, meaning that the envisioned end-users (older mourners) and all other stakeholders (e.g., general practitioners) were at the center of attention when designing and were provided ample space to provide their input. The resulting design consists of a general set-up for the online service, which included Sun, a virtual agent that guides mourners through the main functionalities of the service.

In addition to the generic service, country-specific adaptations were found to be necessary. Getting mourners familiar with the service requires a country-specific approach. And guiding the mourners that need professional support to these professionals also needs a country-specific tactic. So, while the technology can be general, the service model (or implementation plan) accompanying the technology should cater to cultural and geographical differences.

Regarding acceptance, we found that stakeholders (like spiritual counselors or psychologists) have neutral to negative attitudes toward the use of LEAVES by mourners as a stand-alone tool, while they are somewhat positive toward using LEAVES as part of blended counseling. And while mourners who participated in focus groups indicated they are open and willing to try out the online-only intervention, they did mention that online help can never replace traditional, face-to-face help. In these focus groups, participants highlighted the possibility of obtaining a wide range of information, a low starting barrier, anonymity, and independence of time and place as main benefits of using LEAVES. Digital literacy, or the lack thereof, was seen as the main barrier toward use. This is in accordance with a general reluctance toward digital mental health services that was found among a general sample in Australia (March et al., 2018) and in Germany (Phillips et al., 2021), where people preferred face-to-face contact.

Conflicting results were found in the survey for assessing first impressions of the LEAVES service among a wider group of end-users and stakeholders. In the Netherlands and in Portugal, most adults thought they would be able to use LEAVES. In contrast, stakeholders were far more negative on this point, where only around half of the Dutch stakeholders thought that older adults had the necessary skills. In Portugal and Switzerland, about 60% of the stakeholders foresaw problems with digital literacy. Stakeholders’ estimation of digital skills and their preference toward face-to-face over online help might be influenced by their training and a concern of losing clients. Nevertheless, ageism, negative or positive stereotypes on the basis of older age or a perception of persons as being “old” or “elderly” (Iversen et al., 2009) can play a role in the observed pattern of results. Previous studies have found that people have the tendency to dismiss older adult’s digital literacy as problematic, thereby reinforcing older adults’ perceptions of their own digital skills as negative (Arthanat et al., 2019). While the sufficiency of older adult mourners’ digital skills to use LEAVES can only be proven in a real-life evaluation (which is being conducted at expected to be concluded in 2023), for the acceptance of the service the (erroneous) expectation that older adults cannot operate such a service is problematic. Communication about LEAVES toward stakeholders should therefore be accompanied by information about older adults’ competence to use the service, and the possibilities it provides to them for empowering, and closing the digital divide (Hill et al., 2015).

An overview of grief-related digital services and websites by Beaunoyer and colleagues (Beaunoyer et al., 2020) showed that there is a wide variety in the types of support offered online, but that psychoeducation and practical information accounts for about 50% of these online services. Psychoeducation and cognitive-behavioral interventions are one part of LEAVES and the grief support it provides. However, the service also contributes to the state of the art in online grief support by combining psychoeducation and cognitive-behavioral interventions with an initial risk assessment, activity guidance through an ECA, and a biweekly mood checkup. Furthermore, the service model, by being developed alongside the technology itself, facilitates the implementation of the service in the field (Tossaint-Schoenmakers et al., 2021).

Limitations of this study include that the survey that tested the acceptance of LEAVES among stakeholders and end-users was offered online. This digital self-selection strategy could have biased the ratings of older adults’ self-reported digital literacy. Second, how someone deals with grief is to a large extent determined by culture (Moore et al., 2022). Thus, our results are restricted to the three countries in which we collected data (the Netherlands, Switzerland, and Portugal), or other countries with a similar cultural make-up and set of rituals related to mourning. Finally, despite belonging to the target group of older mourners, not all the potential end-users participating in the development of the service were in that grief process. Thus, the conclusions drawn from the assessment of key variables for the acceptance of the service, such as perceived helpfulness and perceived ease of use, may not represent the specific perceptions and usability needs of the older mourners who lost their partner.

Potential technical developments of the LEAVES service could include artificial intelligence algorithms that personalize the presented content of the intervention. In this context, the question may arise to what extent algorithm awareness, that is, the extent to which users are aware of and comprehend the impact of personalization algorithms that covertly run in the background of the service, may affect the target population’s trust in the service, their privacy concerns and propensity to self-disclose and thereby, ultimately, the efficacy of the intervention (Shin et al., 2022).

## Conclusions

The rise of digital self-help services paves the way for a whole range of innovative mental health interventions that allow individuals to deal with mental health or psychosocial problems in their own time, in their own space, and without the mandatory involvement of a trained professional (Taiminen et al., 2018). Although there are significant concerns regarding the digital literacy of end-users (specifically older adults, the target group of LEAVES), coupled with a proclaimed tendency to prefer, in first instance, face-to-face care, a digital landscape of eMental health services will need to be offered soon. The mental health workforce can no longer manage the growing influx of patients and clients, and unlike the rapid proliferation of digital technology, the worldwide shortage of mental health clinicians is expected to remain constant (Olfson, 2016]. Proper development of eMental health interventions (including both technical design as well as service model design) is key toward the success of these innovations. In this article, we have discussed the iterative, human-centered, and stakeholder-inclusive approach we applied in the design of LEAVES as a means to promote other evidence-based eMental health services that can make a difference in people’s lives.

## Supplementary Material

Supplemental Material
