# Supplementary material for: Development of an online service for coping with spousal loss by means of human-centered and stakeholder-inclusive design: the case of LEAVES
Source: Death Stud. 2023 Apr 27;48(2):187–96. doi: 10.1080/07481187.2023.2203680 (PMC11601046; doi:10.1080/07481187.2023.2203680)
Supplement: Supplemental Material [file UDST_A_2203680_SM2233.docx]

Supplementary tables

Table 1. Interview participant demographics.

| **Country** | **N** | **Gender** | **Mean age** | **Time since spousal loss** | **Nature of loss** |
| --- | --- | --- | --- | --- | --- |
| The Netherlands | 10 | Male: 4  Female: 5  Transgender: 1 | 71.4 years | 1-2 years: 2  2-5 years: 4  5-10 years: 2  >10 years: 2 | Unexpected: 6  After long illness: 5 |
| Switzerland | 6 | Male: 3  Female: 3 | 80.0 years | 0.5-1 year: 1  1-2 years: 3  5-10 years: 2 | Unexpected: 1  After long illness: 5 |
| Portugal | 7 | Male: 1  Female: 6 | 74.3 years | 1-2 years: 2  2-5 years: 3  5-10 years: 1  >10 years: 1 | Unexpected: 2  After long illness: 5 |

Table 2. Focus group participants’ characteristics.

| **The Netherlands** | | **Switzerland** | | **Portugal** | |
| --- | --- | --- | --- | --- | --- |
| *Role* | *Gender* | *Role* | *Gender* | *Role* | *Gender* |
| General practitioner | Female | Relative of mourner | Female | Relative of mourner | N/A |
| Psychologist | Female | General practitioner | Male | Geronto-psychiatric nurse | Female |
| Undertaker | Female | Psychologist | Female | Psychologist | Female |
| Social care worker | Female | Association for the elderly | Male | Psychologist | Female |
|  |  |  |  |  |  |
| Municipality representative | Female | Church representative | Female | Psychiatrist | Female |
| Volunteer on program against loneliness | Male | District nurse in psychiatry | Female | Mental health nurse | Female |
| Funeral organization representative | Female | Social worker | Female | Social care worker | Female |
|  |  |  |  | Palliative care nurse | Female |

Table 3. Interview and focus group participants’ demographics.

| **Country** | **N** | **Gender** | **Mean age** | **Time since spousal loss** | **Method** |
| --- | --- | --- | --- | --- | --- |
| The Netherlands | 5 | Male: 2  Female: 3 | 68.8 years | 2-5 years: 2  >10 years: 3 | 1 online focus group |
| Switzerland | 6 | Male: 2  Female: 4 | 74.8 years | 2-5 years: 2  5-10 years: 2  >10 years: 2 | 3 face-to-face interviews, 1 focus group |
| Portugal | 5 | Male: 0  Female: 5 | 76.8 years | <0.5 years: 1  5-10 years: 2  >10 years: 2 | Face-to-face interviews |
